# Urogynecologic Symptoms Are Not Specific to Anatomic Region of Digestive Symptoms

**DOI:** 10.1111/nmo.70081

**Published:** 2025-05-13

**Authors:** Madison Simons, Tatini Sal‐Markar, Debolina Ghosh, Suruchi Ramanujan, Xiao Jing Wang, Yuying Luo

**Affiliations:** ^1^ Digestive Disease Institute Cleveland Clinic Foundation, Department of Gastroenterology, Hepatology, and Nutrition Cleveland Ohio USA; ^2^ Case Western Reserve University School of Medicine Cleveland Ohio USA; ^3^ Divisions of Gastroenterology and Hepatology Mayo Clinic Rochester USA; ^4^ Mount Sinai West & Morningside Mount Sinai Center for Gastrointestinal Physiology and Motility New York New York USA

**Keywords:** DGBI, gender, gynecology, sexual health

## Abstract

**Background/Aims:**

Many patients with digestive symptoms describe underlying urinary or gynecologic symptoms, which may increase visceral sensitivity in the abdominopelvic region and amplify distress around digestive symptoms. The aim of this study was to describe the prevalence of urinary and gynecologic symptoms in patients with disorders of gut–brain interaction (DGBI).

**Methods:**

Consecutive adult patients with a female reproductive tract who were referred for GI behavioral medicine evaluation for DGBI at a tertiary care center between 2022 and 2023 were prospectively evaluated for symptoms associated with urinary and gynecologic dysfunction. Descriptive statistics were conducted to assess the prevalence of these symptoms within this population by anatomic region affected (esophagus, stomach, and bowel).

**Results:**

A total of 432 patients were included in our cohort with a mean (SD) age of 40.0 years [Q1;Q3 29.0–52.0], with a predominantly White population (86.1%). Dysmenorrhea (61.0%) and menorrhagia (58.6%) were commonly reported among women with digestive symptoms. Urinary symptoms were less common, with 18.7% reporting pain with urination, 33.0% reporting difficulty voiding urine, and 26.1% reporting a history of frequent UTIs. 23% of women experienced ≥ 4 urinary/gynecologic symptoms. There were no significant differences in the type of urogynecologic symptoms reported based on the affected area of the GI tract.

**Conclusion:**

Urogynecologic symptoms are common among patients with DGBIs affecting the entirety of the GI tract. We presume the presence of these comorbid symptoms is likely to impact symptom severity, quality of life, and could affect treatment response. Future studies are necessary to understand the mechanisms behind these shared conditions as well as develop effective treatments that address their overlap.

## Graphical abstract

Urogynecologic symptoms are common in patients with DGBIs, regardless of the affected area of the GI tract. We recommend screening for these symptoms in all patients with chronic GI symptoms.

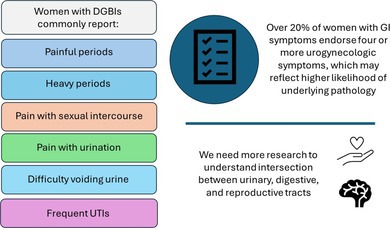

**Summary**

- Urogynecologic symptoms are common in patients with digestive symptoms.
- There are no significant differences in the frequency of these symptoms based on the affected area of the GI tract.
- More research is needed to understand pathophysiological relationships between urogynecologic functioning and GI symptoms.

## 1 Introduction

The presence of refractory gastrointestinal (GI) symptoms should ultimately prompt a treating provider to look for other potential causes outside the GI system. In some cases, diseases affecting other regions of the body may contribute to or amplify GI distress, including those of urinary and gynecologic origin, given the shared dorsal root ganglion connection for the sensory afferent nerves of the bowel, bladder, and reproductive tract [1].

There is some evidence on the overlap between urogynecologic conditions, such as endometriosis and interstitial cystitis (IC), with GI symptoms. While theories regarding pelvic organ cross‐sensitization have been proposed, no definitive pathophysiologic mechanisms have been elucidated to explain the burden of symptoms experienced by patients with these comorbid conditions [2]. Data from a nationwide study demonstrated that patients with irritable bowel syndrome (IBS) are at increased risk of developing IC compared to those without IBS, suggesting the possibility of a shared pathophysiology for these two conditions [3]. The presence of bladder and bowel dysfunction in childhood may be predictive of developing IC and IBS in adulthood [4]. Similarly, over 50% of patients with surgically confirmed endometriosis met criteria for IBS [5]. Lastly, despite just 10%–15% of the general population being affected by endometriosis, 36% of women who met criteria for IBS in one study had endometriosis [6]. While IBS symptoms alone lead to worsened quality of life, distress severity, and treatment refractoriness may be exacerbated by concurrent urogynecologic symptoms, necessitating further investigation on this topic [7].

There is scant literature on the co‐occurrence of urinary and gynecologic symptoms in patients with digestive diseases. Existing studies are limited to those who fall into discrete diagnostic categories (e.g., IC, endometriosis, or IBS). Although this is more objective from a scientific standpoint, it likely does not capture the spectrum and burden of symptoms patients are experiencing; in clinical practice, IC and IBS have largely been considered diagnoses of exclusion, and endometriosis can only be fully confirmed with laparoscopic surgery. Thus, this disregards a large group of patients who experience symptoms but do not have a formal diagnosis.

The primary aim of this study is to investigate the prevalence of urinary and gynecologic symptoms in patients born with a female reproductive tract referred for GI behavioral medicine evaluation. We hypothesize that patients with lower‐GI symptoms will be more likely to report urinary and gynecologic symptoms, given shared visceral cross‐talk between these organ systems. A more robust characterization of clinical phenotypes and increased awareness of the overlap between urogynecologic symptoms and GI distress will help guide more comprehensive treatment approaches.

## 2 Methods

Biological females who were referred for GI behavioral medicine evaluation for a disorders of gut–brain interaction (DGBI) diagnosis at an outpatient GI clinic in a tertiary care center between February and October 2023 were included in this study. Data was collected prospectively by two GI psychologists during the initial evaluation for all patients in a semi‐structured clinical interview. The questions listed in Table 1 represented the starting point for each inquiry regarding the presence of urinary and gynecologic symptoms. The provider would then ask clarifying questions to determine the clinical significance of the patient's response. For example, if a patient endorsed dysuria, but only in the context of a urinary tract infection (UTI), this would be classified as “no” to having this symptom.

**TABLE 1:** Questions used to assess urogynecologic symptoms.

Do you experience painful periods? If you have already gone through menopause, did you previously experience painful periods?
Do you experience heavy menstrual bleeding (soaking through more than one tampon/pad within 2 h)? If you have already gone through menopause, did you previously experience heavy menstrual bleeding?
Do you experience pain with sexual intercourse? If yes, at insertion or deep inside?
Do you experience pain when you pee?
Do you ever have difficulty emptying your bladder?
Have you ever had a history of frequent urinary tract infections?

Other information extracted from the medical record included: demographic information (age and race) and primary GI encounter diagnosis. The primary GI diagnosis is based on the condition for which the patient was referred to GI behavioral medicine by their gastroenterologist. In patients for whom more than one GI diagnosis was present, the primary encounter diagnosis reflects the patient's presenting complaint at the time of the visit. The total sample was divided into three anatomical groups based on the affected area of the GI tract for the primary diagnosis: esophagus (including gastroesophageal reflux, functional heartburn, and reflux hypersensitivity), stomach (including functional dyspepsia and gastroparesis), and bowel (including IBS, functional constipation, and functional diarrhea). Pre‐ versus postmenopausal status was based on an age cutoff of 50 years. This study was approved by the Institutional Review Board of the Cleveland Clinic Foundation.

## 3 Statistical Analysis

Data were described using median and interquartile range (IQR) for non‐normal continuous variables and frequency (percentage) for categorical variables. The Shapiro–Wilk normality test was used to determine the normality of continuous variables. Missing values were still treated as missing. Total variables were created for the frequency of urinary and gynecologic symptoms and reported as categorical variables in the summary tables; numerical count variables were used when assessing correlations. The Pearson's Chi‐square test and Fisher's exact test were used to compare the categorical variables (demographic characteristics and urogynecologic symptoms) among anatomic regions. Chi‐square analysis compared the frequency of urogynecologic symptoms in pre‐ and postmenopausal patients. The Kruskal–Wallis test was used to compare the continuous variables (total number of urogynecologic symptoms) among groups. Analyses were performed using R software, and a significance level of 0.05 was assumed for all tests.

## 4 Results

### 4.1 Sample Characteristics

The final sample included 432 patients. The median age of patients was 40.0 years, and the median BMI was 24.7 kg/m [2]. Our cohort included 86.1% White and 89.4% non‐Hispanic patients (Table 2). There were no differences in demographic characteristics between diagnostic groups. 57% of the sample had an anxiety disorder associated with their billing code, and 6% had a depressive disorder associated with their billing code. 25% of the sample was older than 50 years and considered to be in menopause.

**TABLE 2:** Demographic characteristics by disease group in urogynecologic sample.

	Total *N* = 432	Esophagus *N* = 28	Stomach *N* = 185	Bowel *N* = 157	*p*
Age, median [Q1;Q3]	40.0 [29.0;52.0]	37.5 [29.8, 51.2]	40.0 [28.0;51.0]	41.0 [28.0;56.0]	0.95
BMI, median [Q1;Q3]	24.7 [21.0;30.6]	25.3 [21.0, 29.5]	25.7 [20.9;31.9]	24.0 [21.2; 29.6]	0.28
Race					0.85
White	369 (82.2%)	23 (82.1%)	156 (84.8%)	139 (88.5%)	
Black	18 (4.17%)	1 (3.57%)	1 (3.57%)	5 (3.18%)	
Other	13 (3.00%)	0 (0.0%)	0 (0.00%)	2 (1.27%)	
Unavailable	31 (7.18%)	4 (14.3%)	4 (14.3%)	11 (7.01%)	
Ethnicity					0.81
Non‐Hispanic	373 (86.3%)	24 (85.7%)	164 (88.6%)	141 (89.8%)	
Hispanic	15 (3.47%)	2 (7.14%)	5 (2.70%)	6 (3.82%)	
Unavailable	30 (6.94%)	2 (7.14%)	18 (8.65%)	10 (6.37%)	

Abbreviation: BMI = body mass index, kg/m^2^.

Table 3 provides information about surgical, procedural, and diagnostic history related to urogynecologic function for the total sample. Given the small number of patients within each group, we did not make comparisons between anatomic regions of the GI tract. 11% of the total sample was previously diagnosed with endometriosis, and 3% were diagnosed with IC. Abdominal surgery (10%) and laparoscopy (9%) were the most common surgical interventions, and pelvic ultrasound (10%) was the most common procedure among this sample.

**TABLE 3:** Surgical and procedural history of the total sample.

	Total *N* = 432
Surgical history	
Abdominal surgery	44 (10.11%)
Hysterectomy	16 (3.70%)
Laparoscopy	41 (9.61%)
Colectomy	26 (6.07%)
Appendectomy	4 (1.00%)
Fundoplication	1 (0.16%)
Prior urogyn diagnoses	
Endometriosis	50 (11.64%)
Interstitial cystitis	14 (3.20%)
Procedural history	
Pelvic MRI	6 (3.37%)
Pelvic ultrasound	45 (10.45%)
Cystoscopy	3 (0.67%)
Bladder hydrodistension	2 (0.51%)

### 4.2 Urogynecologic Symptoms

Gynecologic symptoms were commonly reported, with 60% of patients endorsing a history of dysmenorrhea, 56.7% endorsing a history of menorrhagia, and 38.6% endorsing a history of dyspareunia. Urinary symptoms were less commonly reported. However, 16.2% report pain with urination (dysuria), 26.9% endorse a history of difficulty voiding urine, and 24.7% endorse a history of frequent UTIs (Tables 3, 4, 5). Overall, nearly 20% of patients endorse four or more urogynecologic symptoms.

### 4.3 Anatomic Diagnostic Group Breakdown

**FIGURE 1:**
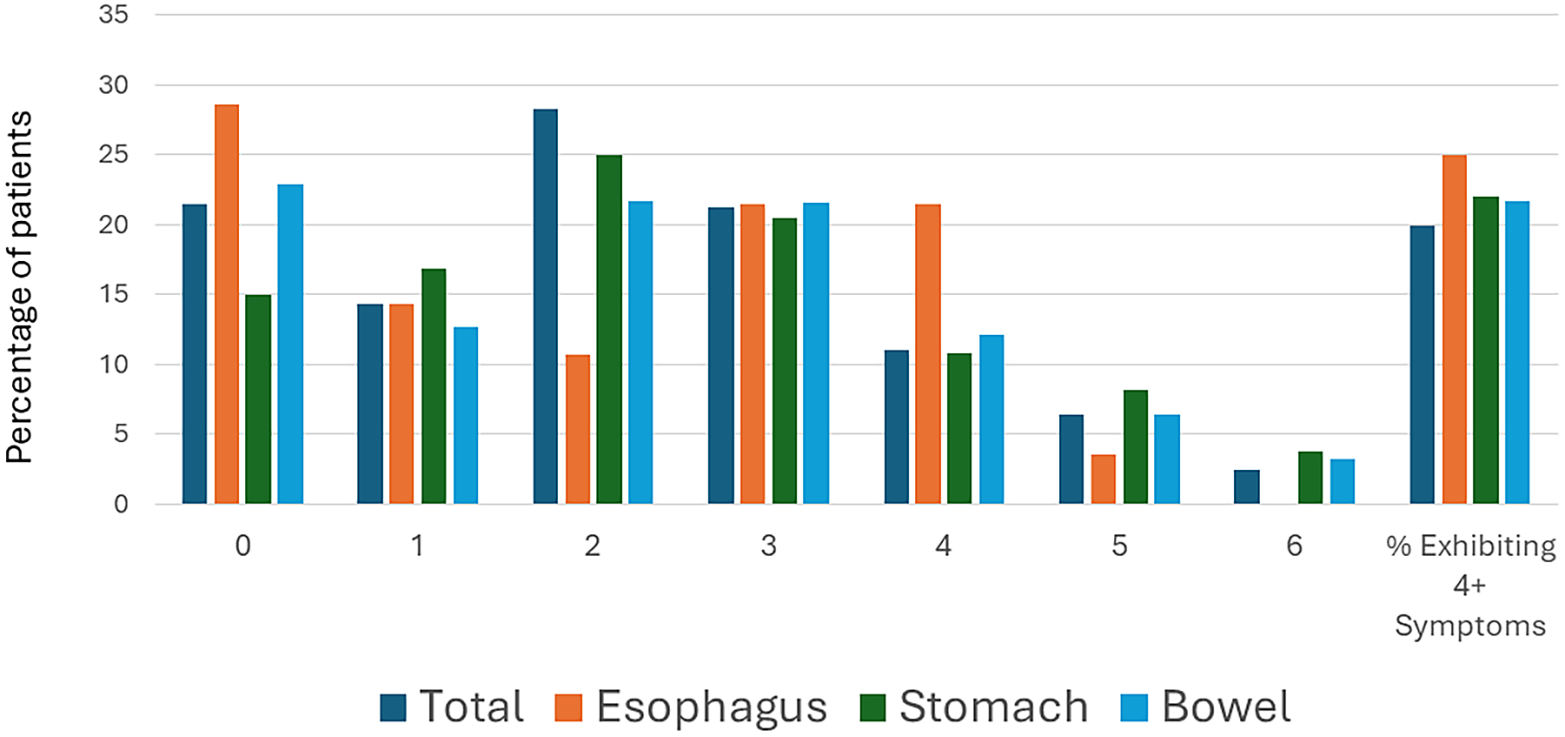
Number of urogynecologic symptoms reported by disease group. Note: Percentage of patients reporting total number of urogynecologic symptoms based on anatomic region of the GI tract.

Broadly, there were no significant differences in the prevalence of urogynecologic symptoms based on anatomic region, including dysmenorrhea (*p* = 0.554), menorrhagia (*p* = 0.076), dyspareunia (*p* = 0.812), and dysuria (*p* = 0.086). Across anatomic regions, patients were likely to report a similar frequency of urogynecologic symptoms (Tables 4, 5, 6), with just over 20% of patients reporting four or more symptoms (Figure 1, Tables 4, 5, 6).

**TABLE 4:** Presence of urogynecologic symptoms by diagnostic group.

	Total *N* = 418	Esophagus *N* = 28	Stomach *N* = 185	Bowel *N* = 157	*p*
Dysmenorrhea	255 (61.0%)	16 (57.1%)	113 (63.1%)	87 (58.6%)	0.554
Menorrhagia	245 (58.6%)	14 (50.0%)	108 (61.0%)	82 (54.3%)	0.076
Dyspareunia	163 (38.9%)	66 (37.1%)	66 (37.1%)	64 (42.4%)	0.812
Dysuria	78 (18.7%)	5 (17.9%)	36 (20.0%)	26 (17.1%)	0.086
Difficulty voiding urine	138 (33.0%)	8 (28.6%)	58 (32.2%)	51 (33.8%)	0.824
History of frequent UTIs	109 (26.1%)	5 (18.5%)	53 (29.9%)	33 (22.6%)	0.061

Abbreviation: UTIs = urinary tract infections.

### 4.4 Symptom Comparison by Pre‐ and Postmenopausal Status

Pre‐ and postmenopausal females differed in difficulty voiding (*p* = 0.02) and frequent UTIs (*p* = 0.001), where postmenopausal females were more likely to report these symptoms. There were no statistically significant differences in dysmenorrhea (*p* = 0.69), menorrhagia (*p* = 0.11), dysuria (*p* = 0.31), or dyspareunia (*p* = 0.07).

## 5 Discussion

This study importantly demonstrates that over half of the women referred for GI behavioral medicine evaluation experience gynecologic symptoms, such as dysmenorrhea and menorrhagia. Over a fifth of women with digestive symptoms additionally report urinary symptoms like frequent UTIs and difficulty voiding urine. Notably, there was no difference in the prevalence of urogynecologic symptoms based on the area of the GI tract affected; those with esophageal symptoms were just as likely as those with stomach or bowel symptoms to report urogynecologic symptoms. Postmenopause was associated with being more likely to report difficulty emptying the bladder and frequent UTIs. The consistency between our findings and existing literature underscores the need for more studies on the underlying relationship between the GI, urinary, and reproductive tracts to shorten time to diagnosis and improve clinical outcomes.

Previous studies have shown substantial overlap between patients who experience digestive, gynecologic, and urologic symptoms [8, 9, 10]. However, this has largely focused on the overlap between urogynecologic symptoms and bowel disorders; there has been little published on the prevalence of these symptoms in patients with upper‐GI symptoms. The current study highlights that patients with symptoms affecting all regions of the GI tract are equally affected by urinary and gynecologic symptoms. In a large network analysis of DGBI symptoms, the authors demonstrate how pain bridged anatomic locations in DGBI (i.e., abdominal pain connected to chest pain, epigastric pain, or rectal pain), and suggest it may be more appropriate to define conditions based on their cardinal symptom (e.g., pain) versus anatomic region [11]. It is worth exploring in the future whether some of the symptoms assessed here reflect alterations in communication between the brain and visceral organs, such as what would be present in a DGBI (e.g., dysmenorrhea, dyspareunia, dysuria, and difficulty emptying the bladder (as an expression of altered bladder sensation)).

Previous literature has shown that 36% of patients with IBS have a concurrent diagnosis of endometriosis, compared to 10% of the general population [6]. Over half of the patients in our study endorsed the presence of multiple gynecologic symptoms. This discrepancy may be because more patients experience gynecologic symptoms than have been formally diagnosed with endometriosis and highlights the need to assess for gynecologic symptoms in patients with chronic digestive symptoms. Although endometriosis can sometimes be detected with imaging (such as a transvaginal ultrasound specifically used to identify adhesions between pelvic organs or an endometriosis‐specific magnetic resonance imaging (MRI) study), the gold standard for diagnosing endometriosis remains laparoscopic surgery, ideally with excision of the endometrial lesions. While excision surgery is helpful for many patients, it does not always eliminate symptoms, especially if there is another comorbid condition such as adenomyosis, and some will undergo repeat procedures, increasing the risk of abdominal/pelvic adhesions. It is not clear how gynecologic treatments, including the use of oral contraceptives, intrauterine devices (IUDs), or surgery (excision surgery or hysterectomy/oophorectomy), affect digestive symptoms in women with and without endometriosis. There is one prior study demonstrating the benefits of linaclotide on endometriosis‐associated vaginal hyperalgesia, suggesting interventions targeting bowel symptoms may also improve quality of life in endometriosis [12].

Similarly, studies have shown that almost half of patients with IBS also experience IC [3]. This is consistent with our study, which found 40% of patients with digestive symptoms also experience urologic symptoms (Table 5). Urinary symptoms were overall less prevalent in our GI patients than gynecologic symptoms, which may relate to the greater proximity of the bowel to the reproductive organs in comparison to the urinary system and overall higher prevalence of gynecologic conditions. Much of the literature on IBS and IC refers back to their shared profile as functional pain conditions [13], with no identifiable pathology to explain the level of distress experienced by the patient.

**TABLE 5:** Total urogynecologic symptoms by affected area of GI tract.

Urogyn total score	Total *N* = 432	Esophagus *N* = 28	Stomach *N* = 185	Bowel *N* = 157
0	84 (19.4%)	8 (28.6%)	28 (15.1%)	36 (22.9%)
1	61 (14.1%)	4 (14.3%)	31 (16.8%)	20 (12.7%)
2	94 (21.8%)	3 (10.7%)	46 (24.9%)	34 (21.7%)
3	92 (21.3%)	6 (21.4%)	38 (20.5%)	33 (21.0%)
4	55 (12.7%)	6 (21.4%)	20 (10.8%)	19 (12.1%)
5	33 (7.63%)	1 (3.57%)	15 (8.11%)	10 (6.37%)
6	13 (3.00%)	0 (0.00%)	7 (3.78%)	5 (3.18%)
% Exhibiting 4+ urogynecologic symptoms	23.33%	24.97%	22.69%	21.65%

**TABLE 6:** Breakdown of urinary and gynecologic symptoms by disease group.

	Total *N* = 575	Esophagus *N* = 28	Stomach *N* = 185	Bowel *N* = 157
Total gynecologic symptoms				
0	153 (27.2%)	10 (35.7%)	43 (23.9%)	44 (28.8%)
1	94 (16.7%)	4 (14.3%)	32 (17.8%)	26 (17.0%)
2	176 (31.3%)	7 (25.0%)	60 (33.3%)	42 (27.5%)
3	139 (24.7%)	7 (25.0%)	45 (25.0%)	41 (26.8%)
Total urinary symptoms				
0	317 (56.2%)	15 (53.6%)	88 (48.4%)	80 (52.3%)
1	152 (27.0%)	8 (28.6%)	56 (30.8%)	46 (30.1%)
2	63 (11.2%)	5 (17.9%)	23 (12.6%)	17 (11.1%)
3	32 (5.67%)	0 (0%)	15 (8.24%)	10 (6.54%)

Understanding the overlap between digestive and urogynecologic symptoms is crucial because multiorgan involvement may exacerbate symptom severity. A 2005 study connected the severity of GI symptoms to the spread of endometriosis in the bowel, suggesting a link between increased severity of gynecologic and digestive symptoms [14]. On the other hand, a 2018 study investigating IBS symptom severity in patients with endometriosis found increased symptom severity in patients with less severe endometriosis than in patients with more severe endometriosis [15]. While our data did not investigate symptom severity, the equivocality of the current literature points to a need for a better understanding of the interplay between urogynecologic symptoms and GI symptom severity.

The consequences of overlapping pelvic and GI pain conditions are substantial, including potential diagnostic delays, increased healthcare utilization, and increased risk of invasive procedures. IBS alone has been associated with increased medical cost burden for both individuals and society, one major cause of which is diagnostic investigation [16, 17]. In another study, the authors showed that the presence of multiple functional somatic syndromes (FSSs), new onset chronic pelvic pain, and new onset IBS was associated with increased likelihood of hysterectomy in women with bladder pain syndrome/IC (13) [18]. Recognizing how frequently patients concurrently experience GI, gynecologic, and urologic symptoms is the first step in better characterizing the nature of this overlap and identifying patients at risk before costly and invasive diagnostics and procedures. It is worth noting in this sample that, despite the high rates of reported urinary and gynecologic concerns, a relatively low number of patients have undergone diagnostic procedures (such as MRI, ultrasound, and cystoscopy) to explore these concerns further.

While the mechanisms behind the overlap in digestive and urogynecologic symptoms are relatively underexplored, some evidence highlights the roles of inflammation, altered pain sensitivity, and cross‐sensitization pathways, damage to the interstitial cells of Cajal, and increased estrogen. Chronic inflammation may lead to increased intestinal permeability and mast cell activation, leading to nociceptive pathway activation [19]. It is also possible that endometriotic tissue activates sensory neural pathways in the bowels, resulting in a decreased pain threshold in the GI tract [20]. Experiencing GI and urogenital symptoms may also be secondary to overlapping sensory neural networks [21]. A systematic review from 2022 suggests that the overlap between IBS and endometriosis symptomatology may stem from female sex hormones like estrogen [22]. Future research should investigate the complex underlying pathophysiology and opportunities for intervention in patients experiencing overlapping abdominopelvic pain.

Strengths of this study include the large sample size of patients and comprehensive spectrum of gynecologic and urologic symptoms assessed prospectively in a range of digestive conditions (rather than focusing on specific diagnoses such as endometriosis and IC).

## 6 Limitations

Limitations include a cross‐sectional study design and a racially homogenous sample. In addition, the design of this study potentially introduces bias into the results, both in how a patient interpreted the questions that were asked as well as whether the provider deemed the patient's responses to be of clinical significance (enough to endorse “yes” to a particular symptom). Several of the symptoms assessed here cannot be ascertained objectively, including the amount of pain associated with the menstrual cycle, so this is subject to the patient's report of their experience. In addition, there is no definitive endpoint for how many UTIs or in what exact frequency would be classified as having had frequent UTIs; however, the presence of recurrent UTIs has been associated with a higher likelihood of endometriosis [23]. Future studies using visual analog scales may be able to better delineate the severity of these symptoms and provide a sense of objectivity to constructs that may be challenging to measure.

Because symptoms were ascertained at initial patient evaluation, it is possible that later symptoms were unreported, leading to an underestimation of symptom prevalence. Similarly, urogynecologic symptoms can fluctuate significantly over the course of the menstrual cycle and the female lifespan (such as in menopause). The stage of the menstrual cycle was not assessed in this study, and therefore, we are not able to draw more specific conclusions on the relationship between the menstrual cycle, digestive symptoms, and hormone levels. Postmenopause was associated with a higher likelihood of reporting urinary symptoms, including difficulty emptying the bladder and frequent UTIs, which is consistent with existing literature [24]. We recommend more rigorous prospective analyses in the future aimed at evaluating these relationships, including how hormone fluctuations may influence the development and exacerbation of digestive symptoms.

Other factors may influence the presence of urogynecologic symptoms that have not been reported here, including a previously known diagnosis of a urinary or gynecologic condition (e.g., endometriosis, IC, and polycystic ovarian syndrome), use of contraceptive medications or hormone replacement therapy, or a history of sexual trauma. In addition, this study may not be representative of the full spectrum of urogynecologic conditions. We focused on the six symptoms assessed here as those with a higher likelihood of predicting endometriosis [23]. However, we also recognize there are other urinary and gynecologic symptoms that have not been assessed here, including urinary frequency, generalized pelvic pain, nycturia, and urinary incontinence.

We did not differentiate between Rome IV bowel and anorectal disorders. For this study, we used the primary GI diagnosis associated with the encounter to make disorder classifications, and there were not sufficient patients with primary anorectal conditions (*n* = 4) to justify making a separate category to make analysis feasible with the amount of comparisons being made. Although it could perhaps have been extrapolated that those for whom dyssynergia is a contributing factor could also include anorectal manometry (ARM) data, this would have biased our results, as ARM is typically only ordered in patients for whom dyssynergia is suspected. For these reasons, and given the frequency at which DGBIs overlap [25], we included anorectal disorders as part of the bowel region. Given our practice in a large tertiary care center, most patients included in this sample experience symptoms associated with multiple GI conditions, often spanning multiple anatomic regions. A recent systematic review demonstrated the significant overlap between DGBIs, where 36.5% of patients experienced symptoms of more than one DGBI [25]. In addition, the most recent Rome Working Team report highlights that “organic” GI conditions also commonly overlap with DGBIs [26]. These highlight further that the particular anatomic region of the GI tract, or even whether it is considered “functional” or “organic,” may be less important than the cardinal symptom (e.g., pain) that the patient experiences.

It is also important to recognize the population reflected in this study, conducted at a specialized tertiary care center, which may not be generalizable to all GI clinics. Our existing model is such that, ideally, all patients will be seen by the gastroenterologist and behavioral medicine provider concurrently throughout treatment, using a medical home model. However, it is often the case that those referred for behavioral medicine evaluation may have symptoms that are refractory to other interventions that have been previously tried. Thus, the patients included in this sample may represent a higher level of complexity/acuity than a general GI patient population. Although we included anxiety and depression diagnoses within this sample, given they were referred for behavioral medicine evaluation, these numbers are not likely to be accurate depictions of the patients' psychological state, as they are subject to errors in diagnosis and coding that are prevalent in studies based on EMR review and not based on objective data.

Finally, this study did not explore the overall prevalence of urogynecologic symptoms in matched populations without GI conditions, nor the pathophysiology of overlapping digestive and urogynecologic symptoms. Population‐based prevalence rates of urinary and gynecologic symptoms, to compare with the data collected in this study, are difficult to estimate given highly heterogeneous samples. For example, the prevalence of dysmenorrhea reported in prior studies is between 16% and 91%, with college students reporting the highest rates of dysmenorrhea [27]. Future studies are needed to better quantify these comorbidities and eventually correlate clinical screening tools with urogynecologic functioning.

## 7 Conclusion

Our study highlights the considerable overlap between digestive and urogynecologic symptoms and more robust clinical phenotypes in this population. Although we have classically thought of visceral organ cross‐talk as it relates to the abdominopelvic region (as in IBS), this study indicates it is important to assess for gynecologic symptoms across chronic GI symptoms affecting other areas of the GI tract as well. Awareness of this overlap in patients with GI symptoms may facilitate appropriate referrals and treatment pathways that can attenuate symptom severity and improve patient outcomes. While the exact mechanism underlying the concurrence of GI and urogynecologic symptoms remains incompletely understood, there is a need for future research investigating pathophysiology and potential avenues for intervention.

## Author Contributions

M.S. contributed to study design, data collection, and interpretation, drafting the initial manuscript. S.R., T.S.M., and D.G. contributed to drafting the initial manuscript. X.J.W. and Y.L. contributed to the study design and data collection/interpretation. All authors provided review and approval of the final manuscript.

## Conflicts of Interest

The authors declare no conflicts of interest.

## Data Availability

The data that support the findings of this study are available from the corresponding author upon reasonable request.
